# Expression Patterns of *HvCKX* Genes Indicate Their Role in Growth and Reproductive Development of Barley

**DOI:** 10.1371/journal.pone.0115729

**Published:** 2014-12-22

**Authors:** Wojciech Zalewski, Sebastian Gasparis, Maja Boczkowska, Izabela K. Rajchel, Maciej Kała, Wacław Orczyk, Anna Nadolska-Orczyk

**Affiliations:** 1 Department of Functional Genomics, Plant Breeding and Acclimatization Institute – National Research Institute, Radzikow, Blonie, Poland; 2 Department of Genetic Engineering, Plant Breeding and Acclimatization Institute – National Research Institute, Radzikow, Blonie, Poland; Institute of Genetics and Developmental Biology, Chinese Academy of Sciences, China

## Abstract

Cytokinin oxidase/dehydrogenase proteins (CKX) are encoded by a multigene family of *CKX* genes with a varying number of members depending on species. For some of the genes, spectacular effects on grain production in selected cereals have been observed. Despite the fact that partial or full length sequences of most *HvCKX* genes in barley (*Hordeum vulgare*) have already been published, in most cases their specific biological functions have not been reported. Detailed expression patterns for five *HvCKX* genes in different organs/tissues of developing barley plants coupled with analysis of RNAi silent for two genes are presented to test the hypothesis that these expression profiles might indicate their function. Elevated expression for four of them – *HvCKX1*, *HvCKX9*, *HvCKX4*, and *HvCKX11* – was found in developing kernels of wild-type plants compared to other tissues. *HvCKX5* was mainly expressed in leaf tissue. Lower expression was noted for *HvCKX1* in seedling roots and for *HvCKX9* in leaves. The documented effect of RNAi silencing of *HvCKX1* and a trend for *HvCKX9* was higher plant productivity, and the trait was inherited through four generations. Higher plant yield was determined by higher numbers of seeds and spikes. Increased productivity was significantly greater in *HvCKX1* silenced plants showing higher relative expression of *HvCKX1* in developing kernels of wild-type plants compared to the expression of *HvCKX9*. Both *HvCKX1* silenced T_1_ seedlings of cv. Golden Promise and the newly transformed breeding line STH7308 showed greater root mass, but this trait was not inherited in the next generation. Similarly *HvCKX9* silenced T_1_ seedlings exhibited greater plant height without inheritance in the next generation. It is suggested that these effects were not inherited because of compensation by other genes co-ordinately regulating reproductive development. One line with untypically changed, inherited phenotype, which was selected from several dozen silenced lines showing stable and common phenotypes is presented.

## Introduction

Cytokinins are important plant hormones that regulate a number of developmental and physiological processes during plant development. They control root growth and branching, leaf expansion, chloroplast formation, delay of senescence, seed germination [1], [2], maintenance of shoot meristem function [3], metabolic modulation and morphogenesis in response to environmental factors [4], [5], nutritional signaling [6], activity of reproductive meristems and seed yield in cereals [7]–[10] and *Arabidopsis thaliana* [11], crown root formation [12] and various other functions.

The level of biologically active cytokinins depends on the ratio of cytokinin synthesis and catabolism. Cytokinin biosynthesis is catalyzed by isopentenyl transferase enzymes (IPT), cytokinin degradation by cytokinin dehydrogenase enzymes (CKX), reversible inactivation by zeatin *O*-glucosyltransferases (ZOG) and reactivation by β-glucosidases (GLU) [4], [5], [13]–[15]. The CKX enzymes play possibly the principal role in regulating cytokinin levels in plant tissues [2], [4], [13]. The enzymes irreversibly catalyze cytokinin degradation by selective cleavage of unsaturated isoprenoid side chains into adenine/adenosine moiety. The CKXs differ in their catalytic properties, subcellular localization and expression domains.

The CKX enzymes are encoded by a family of *CKX* genes, varying in number depending on the species. The total number of *HvCKX* genes in barley is not known, but many have been sequenced and partly characterized [16]–[18]. The cloning of the full coding sequence of *HvCKX2*, later annotated as *HvCKX9* [17], was described by Galuszka et al. [16]. Expression of *HvCKX2* in the heterologous host plant showed a cytokinin-deficient phenotype characterized by an enhanced root system and very slow shoot development. Wide genomic studies of *CKX* genes of the *Poaceae* were performed by Mameaux et al. [17]. The authors identified ten of the eleven *CKX* genes predicted to be present in barley by comparative analyses. Two of them, *HvCKX2*.1 and *HvCKX2*.2, were characterized with comparative analysis at the DNA, protein and genetic/physical map levels.

The expression of *CKX* genes is tissue and developmentally specific [19]. Detailed analysis of expression profiles of selected *HvCKX* and *TaCKX* during plant development suggests specialized functions adapted to certain organs [8]–[10], [20]. Lack of known knock-out mutants of these genes in barley is the main barrier to more detailed characterization of their biological functions. One possibility to lower the transcript level of a selected gene or group of homologous genes is to silence their expression by RNA interference (RNAi) technology, as has already been documented for *HvCKX1* [8] and *HvCKX2* [9], now annotated as *HvCKX9* [17]. The expression profiles in developing plants correlated with the effect of silencing of expression of the genes at molecular, biochemical and phenotype levels. Lower CKX activity in *HvCKX1* silenced lines led to higher plant yield and greater root weight and in *HvCKX9* silenced lines to higher productivity. Similarly, reduced expression of *OsCKX2*, which is close homologue of *HvCKX1* [17] caused cytokinin accumulation and increased number of reproductive organs in rice resulting in enhanced grain yield [7]. However overexpression of selected *AtCKX* gene and elevated CKX activity in *Arabidopsis* and tobacco was found to reduce growth of shoots and enhance growth of roots, what supported the hypothesis that cytokinins had opposite, regulatory functions in root and shoot meristems [3], [16].

Here we continue to address the hypothesis that the level and the pattern of expression of a defined *HvCKX* gene might determine the specific phenotype and indicate its function in barley. It has already been shown that silencing of *HvCKX1* and *HvCKX9*, both normally having increased levels of expression in developing kernels of wild-type plants, resulted in higher productivity [8], [9]. This type of research requires coupling an analysis of spatial and temporal expression profiles with the characterization of silenced (by RNAi technology) lines with the target gene.

We examined expression profiles of *HvCKX1*, *HvCKX4*, *HvCKX5*, *HvCKX9* (former *HvCKX2*) and *HvCKX11*, provide new data on silencing of *HvCKX9*, and continue detailed analysis of phenotypic data of *HvCKX1* silenced lines over four generations, to determine the stability of inheritance.

## Materials and Methods

### Plant material and transformation experiments

All experimental material was collected from two spring barley cultivars, Golden Promise and Scarlett, and one breeding line, STH7308, originating from Plant Breeding Strzelce Ltd., Co. The plants were grown in a growth chamber under controlled environmental conditions with 18/15°C day/night temperatures and 16 h photoperiod. The light intensity was 350 µmol ⋅s^−1^ ⋅m^−2^. Six seeds of each line were planted into 17 cm×23 cm×17 cm pots filled with Aura substrate for sowing and bedding out (Hollas Ltd.). Plants were irrigated twice a week and fertilized once a week with multicomponent soil fertilizer Florovit [21] according to the manufacturer's instructions.

*In vitro* culture and transformation experiments were performed with immature embryos of cv. Golden Promise and breeding line STH7308 according to the procedures described by Przetakiewicz et al. [22] and Zalewski et al. [8] with modification. Two-day pre-culture media contained 3 mg l^−1^ dicamba instead of picloram and 2,4-D. The same growth regulator was used in the next medium.

Both genotypes were transformed with the hpRNA type of silencing cassettes cloned into the pMCG161 [23]. The T-DNA of the vector contained the *bar* selection gene under the control of the Ubi1 intron promoter. Immature embryos of Golden Promise were transformed with the *HvCKX9* silencing cassette. Construction of the cassette and the vector was described by Zalewski et al. [9]. T_0_ and T_1_ transgenic lines of Golden Promise expressing *HvCKX1* silencing were selected and described by Zalewski et al. [8]. Their T_2_ to T_4_ generations were developed by self-pollination. Immature embryos of STH7308 were transformed with the same *HvCKX1* silencing cassette that was constructed for Golden Promise transformation in Zalewski et al. [8].

### PCR analysis of transgenic plants

Genomic DNA was isolated from young leaves according to the modified CTAB procedure [24]. PCR reactions were carried out in a 25 µl reaction mixture containing 1× PCR buffer, 2 mM of MgCl_2_, 0.2 mM of dNTPs, 0.4 µM of each primer, 1 U of Platinum Taq polymerase (Life Technologies), and 120 ng of template DNA. Integration of the T-DNA construct in transgenic plants was examined by amplification of different fragments of T-DNA with six pairs of primers. Three pairs of pM primers were used to check the integrity and orientation of the silencing cassettes: pM1,2 and pM3,4 were used for plants transformed with pMCG/HvCKX1 and pM5,6 for plants transformed with pMCG/HvCKX2.

Three pairs of OCS primers primed the amplification of various fragments of OCS terminator from silencing cassette. Each plant was tested with at least three pairs of primers. The primer sequences and PCR reaction conditions were described previously by Zalewski et al. [8].

### Quantitative RT-PCR

Total RNA was isolated from 3-day old seedlings, roots from 5-day old seedlings, meristems from 5-day old seedlings, leaves from 5-day old seedlings, developing and fully developed leaves from 3–4 week old plants, inflorescences 3–4 cm and 6–8 cm long, and developing kernels from spikes 0 days after pollination (DAP), 7 DAP and 14 DAP. For all of the tissues, the TRI reagent (Ambion) was used and the extraction was performed according to the manufacturer's protocol. For developing kernels, it was modified by adding an initial SDS extraction step (Prescott and Martin 1987). Isolated RNA was treated with DNase I Recombinant (Roche). Each time, 500 ng of pure RNA was used for cDNA synthesis using the RevertAid First Strand cDNA Synthesis Kit (Fermentas). For a quantitative PCR assay, four primer pairs were designed for target genes, i.e. for *HvCKX1* qCKX11 5′-TCGTCGTCTACCCACTCAACAAATC-3′ and qCKX12 5′-TTGGGGTCGTACTTGTCCTTCATC-3′; for *HvCKX9* qCKX91 5′-GGCGAACTCTGGATAAATGTCTTG-3′ and qCKX92 5′-AGTTCTGTTCTGGTGAGCAAGTGAC-3′; for *HvCKX4* HC4b1 5′-CCTGAAGTTACTCTCTGCCATTG-3′ and HC4b2 5′-TACAGCAGGCTGACCTTTAACTC-3′; for *HvCKX5* HC5b1 5′-GACCGCTTAACATGACATTCAG-3′ *and HC5b2 5′-CATTCCTCTGTACATCACCAACC-3′; for* *HvCKX11* HC111 5′-CAAGACCTACTTCCCGCACTAC-3′ and HC112 5′-CTTATGTTGTGGATGGATCGAG-3′. The beta-actin gene was used as a reference [25] and primer sequences used for amplifications were as followed: qAct1 5′-AGCAACTGGGATGACATGGAG-3′ and qAct2 5′-GGGTCATCTTCTCTCTGTTGGC-3′. All real-time PCR reactions were performed in a Rotor-Gene 6000 (Corbett) with 1x Sso Fast EvaGreen (Bio-Rad), 0.4 µM primers, and 1 µl cDNA in a total volume of 15 µl. The reactions were carried out at the following temperature profile: 95°C–10 min (95°C–30 s; 60°C–30 s; 72°C–30 s) ×45 cycles; 72°C–2 min, with melting curve at 70–95°C–5 s per step. The relative expression of *HvCKX* genes was calculated according to the ΔΔCt method [26] using beta-actin as a reference. The control plants, transformed with the empty vector pMCG161, were used as a calibrator with expression equivalent to 1.

### Analysis of cytokinin dehydrogenase activity

The enzyme activity was measured according to Frébort et al. [27]. Samples were ground to a fine powder with liquid nitrogen. The powdered material was suspended in a double excess (v/w) of extraction buffer containing 0.2M TRIS-HCl, 1 mM phenylmethylsulfonyl fluoride (PMSF) and 0.3% Triton X-100, pH 8.0. The root or leave extract was then incubated with McIlvaine buffer containing 0.2 M Na_2_HPO_4_, 0.1 M citric acid, 0.23 mM dichlorophenolindophenol (DCIP) and 0.1 mM N6-isopentenyl adenine for 16 h at 37°C. The reaction was stopped by adding 0.3 ml 40% trichloroacetic acid (TCA) and 0.2 ml 2% PAF solution (4-aminophenol in 6% TCA). The product concentration was determined spectrophotometrically at the wavelength 300–700 nm. The total protein concentration was estimated based on the bovine serum albumin (BSA) standard curve according to Bradford [28] procedure.

### Analysis of phenotypic traits

Grains from each tested plant were counted and weighed. 1000 grain weight was calculated by multiplying mean grain weight by 1000. Mass of the roots was estimated in 5-day old seedlings, germinated in Petri dishes on wet glass pearls. The roots from each plant were cut 3 mm from the base, dried on blotting paper and weighed, and then frozen immediately with liquid nitrogen to perform further analyses. The seedlings continued their growth developing new roots for four more days. At least 6 PCR positive plants were tested in each line.

Plant height was measured in mature plants previously removed from the soil.

The measurements of four traits associated with productivity (1000 grain weight, grain number, grain yield and spike number) were performed in four to five subsequent generations: T_0_, T_1_, T_2_, T_3_ and T_4_.

Statistical analysis was done using the Statistica (StatSoft) program. An analysis of variance for normality was calculated using the Shapiro-Wilk test. Student's tests were used to compare values obtained for silenced and wild-type lines and determine which mean values were significantly different at the level P<0.05. Some data were additionally verified by Mann-Whitney U test.

## Results

### Barley *HvCKX* gene family

Thirteen accession numbers of nucleotide sequences of putative members of the *HvCKX* family were identified in the NCBI database (Table 1). For seven of them complete coding DNA sequences (CDS) are available. The length of *HvCKX* ORF ranged from 1432 (*CKX4*) to 1701 (*CKX2.1*) bp. The length of partial CDS ranged from 699 to 1842 bp. The coding sequences of all *HvCKX* genes contain introns with numbers varying from one to five. The *HvCKX* proteins had two typical FAD and cytokinin-binding domains, which are specific to *CKX* family members. Pairwise BLAST indicated that AF490591 accession (*CKX2*) showed 99% homology to JF495484 (*CKX9*). However, no significant homology to *CKX2.1* and *CKX2.2* (JF495488 and JF495489, respectively) was found. 99% homology was also identified between accessions JF495482 (*CKX5*) and AK370106 (cDNA sequence for predicted protein); therefore the gene name should be the same. Summing up, there are eleven different CDS/members of the *HvCKX* family and for seven of them the complete CDS is available.

**Table 1:** Sequence data of barley *HvCKX* gene family members published in NCBI databases.

Gene	Acc. number	Genomic (bp)	cDNA (bp)	Exons	Protein	CDS	References
*CKX1*	JF495479	2320	917	2	305	partial	[17]
*CKX2* a	AF490591	1954	1576	5	526	complete	[37]
*CKX2.1*	JF495488*	4060	1701	3	567	complete	[17]
*CKX2.2*	JF495489*	3278	1692	3	564	complete	[17]
*CKX3*	JF495480	3165	1222	4	408	partial	[17]
*CKX4*	JF495481	1920	1432	5	477	complete	[17]
*CKX5* b	AK370106	NI	1598		532	complete	[18]
*CKX5*	JF495482	3952	1842	4	614	partial	[17]
*CKX7*	JF495483	1601	1601	1	533	complete	[17]
*CKX8*	JF495487**	1264	830	4	227	partial	[17]
*CKX9*	JF495484	1954	1576	5	526	complete	[17]
*CKX10*	JF495485	1531	1391	2	464	partial	[17]
*CKX11*	JF495486	1319	699	3	233	partial	[17]

a - annotated by Mameaux et al. [17] as *HvCKX9* (99% homology to *HvCKX2*)

*- located on chromosome 3H

** - located on chromosome 2H

b - predicted *HvCKX* gene name

NI – not identified. Analyzed genes are underlined.

### Expression profiles of selected *HvCKX* genes in different tissues and organs of barley

**Figure 1:**
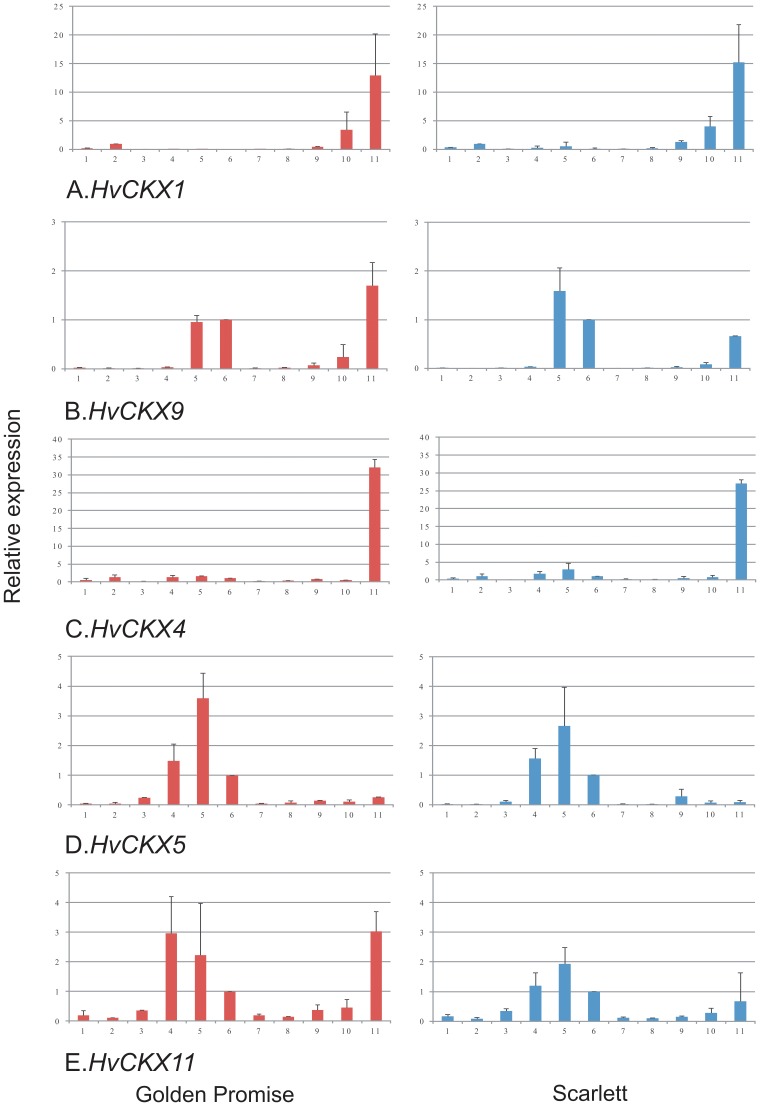
Quantitative analysis of expression patterns of *HvCKX* genes. (A) *HvCKX1*, (B) *HvCKX9*, (C) *HvCKX4*, (D) *HvCKX5*, (E) *HvCKX11* expression in different tissues and organs of developing plants of two barley cultivars, Golden Promise (left side) and Scarlett (right column). Total RNA was extracted from: 1) 3-day old seedlings, 2) roots from 5-day old seedlings, 3) meristems from 5-day old seedlings, 4) leaves from 5-day old seedlings, 5) developing leaves from 3–4 week old plants, 6) fully developed leaves from 3–4 week old plants, 7) inflorescences 3–4 cm long, 8) inflorescences 6–8 cm long, 9) developing kernels 0 DAP, 10) developing kernels 7 DAP, 11) developing kernels 14 DAP. The level of expression was related to the root  = 1.00 (A) or fully developed leaves  = 1.00 (B,C,D,E).

A quantitative analysis of expression of *HvCKX1*, *HvCKX9*, *HvCKX4*, *HvCKX5* and *HvCKX11* genes was performed in different tissues of developing wild-type plants of Golden Promise and Scarlett (Fig. 1a,b,c,d,e). The analyzed tissues/organs were: 3-day old seedlings, roots from 5-day old seedlings, meristems from 5-day old seedlings, leaves from 5-day old seedlings, developing and fully developed leaves from 3–4 week old plants, inflorescences 3–4 cm and 6–8 cm long, and developing kernels from spikes 0 DAP, 7 DAP and 14 DAP. The expression level of *HvCKX1* in these tissues is relative to that in seedling roots which is assumed to be 1.0. In the case of *HvCKX9*, *HvCKX5*, *HvCKX11* genes, a value of 1.0 was assumed for the fully developed leaf stage and this was used as a reference for estimation of relative expression level in the rest of tissues/organs.

Expression of *HvCKX1* (JF495479) was increasing in developing kernels from 0 DAP through 7 DAP to 14 DAP significantly in both cultivars (Fig. 1a). Relative data increases from about 0.5 to 12.9 in cv. Golden Promise and from 1.3 to 15.3 in cv. Scarlett. Expression was also visible in seedling roots, where it was estimated at a relative level of ca. 1.0. There was no transcript of the gene or it was at a very low level in the leaves and meristems of 5-day old seedlings and inflorescences.

*HvCKX9* (JF495484), former *HvCKX2* (AF490591) expression was visible in developing and fully developed leaves at a relative level of ca. 1.0 (in relation to fully developed leaves) and was increasing in developing kernels from 0.3 to 1.7 in Golden Promise and from 0.3 to 0.7 in Scarlett (Fig. 1b).

Expression of *HvCKX4* (JF495481) was apparent in almost all tissues and organs (Fig. 1c). The lowest transcript level occurred in meristems from 5-day old seedlings as well as in inflorescences in both cultivars (0.1 to 0.3), and was much higher in fully developed, developing or seedling leaves, ranging from 1.0 to 1.74 in Golden Promise and from 1.0 to 3.0 in Scarlett. The transcript level rose to 32.2 in 14 DAP kernels of cv. Golden Promise and to 27.0 in cv. Scarlett.

The transcript of *HvCKX5* (AK370106) gene was mainly visible in the leaves, with the level increasing from 1.0 in fully developed leaves through about 1.5 in seedlings to the highest level in developing leaves (3.6 in cv. Golden Promise and 2.7 in cv. Scarlett) (Fig. 1d).

*HvCKX11* (JF495486) expression was the highest in the leaves and 14 DAP kernels in both cultivars (Fig. 1e). The pattern and the level of expression of *HvCKX11* were similar to *HvCKX5*, excluding 14 DAP kernels.

Generally, the patterns of expression in different tissues/organs were very similar in both cultivars for all tested *HvCKX* family members.

### Selection of transgenic Golden Promise lines expressing a silencing cassette for *HvCKX9* and evaluation/estimation of phenotype

*Agrobacterium*-mediated transformation of cv. Golden Promise with a cassette for *HvCKX9* silencing resulted in selection of 31 pMCG/CKX9 transgenic lines. Additionally, 5 transgenic lines transformed with the same T-DNA but without the silencing cassette (pMCG161) were obtained as a control. The mean transformation efficiency was 3.47 (±2.82) and these 36 selected transgenic lines showed a common and stable phenotype. The results with 7 preliminary lines examined up to the T_1_ generation were previously presented to compare two basic methods of cereal transformation [9]. Here we continue presentation of new results with 16 lines selected in T_1_ and 25 lines selected in T_2_ and more advanced generations.

**Figure 2:**
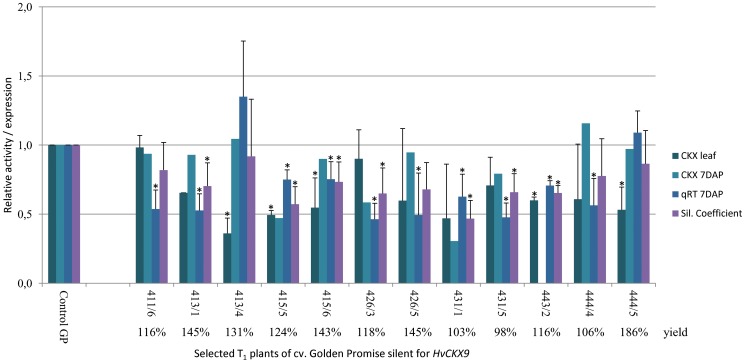
An example of characteristics of segregating T_1_ plants of cv. Golden Promise silent for *HvCKX9*. Bars represent relative levels of CKX activity in leaves and 7 DAP spikes, quantitative transcript level in 7 DAP spikes, and silencing coefficient. Grain yield was estimated as a percentage compared to 100% of control GP. The numbers below the columns are transgenic lines: T_0_/T_1_ plant. * - significant differences at *P*<0.05.

Segregating plants from lines of T_1_ and T_2_ generations were characterized including enzyme activity in developing kernels 7 DAP or 14 DAP and/or leaves as well as transcript levels at 7 DAP or 14 DAP. The silencing coefficient was designated as the mean of a trait. An example of characteristics of selected plants of T_1_ is shown in Fig. 2. Plants were selected for evaluation in subsequent generations based on the silencing coefficient together with the yield of segregating plants.

### Selection of transgenic STH lines expressing a silencing cassette for *HvCKX1*

The STH7308 breeding line was selected from among 20 other breeding lines and 24 cultivars tested for its regeneration capacity *in vitro* (not shown). The line was used for *Agrobacterium*-mediated transformation experiments with the cassette for *HvCKX1* silencing. From 713 explants inoculated by *A. tumefaciens* 44 regenerating callus lines were selected on phosphinothricin with a mean of 2.49 (±1.58) plants per line. At least one PCR positive plant was found in each tested line, with the mean transformation efficiency 16.2%. Transgenic plants from this new genotype were used to test whether a positive correlation existed between CKX activity in seedling roots of STH7308 plants silent for *HvCKX1* T_1_ and mass of roots (results described below).

### Testing the link between pattern of expression and phenotype

In the first experiment we have been tested if decreased activity of CKX enzyme in the leaves of T_1_ plants silent for *HvCKX9* leads to increased plant height and grain yield.

Activity of CKX enzyme was measured in developing as well as developed leaves from 48 T_1_ plants with the silenced *HvCKX9* gene. The plants were divided into three groups with the mean relative enzyme activity of 0.7 (±0.2), 0.8 (±0.1) and 1.0 (±0.1). These data were negatively correlated with plant growth and grain yield (Table 2). The greatest growth of plants, 64.1 cm (±2.1), and grain yield, 3.8 g (±1.1), was obtained in the group exhibiting the least enzyme activity. Plants expressing the greatest CKX activity, in comparison to control, were only 55.7 cm high, with a mean grain yield of 2.6 g. Mean data of plant height in 9 of 10 tested T_1_ lines (six plants each) were significantly greater than in control lines (5 lines ×6 plants). These results indicate that decreased CKX activity in the leaves of T_1_ plants would lead to increased plant height and grain yield.

**Table 2:** Comparison of means of CKX enzyme activity with the plant height and grain yield in plants silent for *HvCKX9* T_1_.

Number of plants from 10 T_1_ lines	Range/mean of CKX activity (st. dev.)			Plant height (cm)	Grain yield (g)
	developing leaf	developed leaf	mean		
35	0.19–1.05/0.7 (±0.2)	0.33–1.15/**0.7 (±0.2)**	**0.7 (±0.2)**	**64.1 (±2.1)**	**3.8 (±1.1)**
17	0.35–1.16/0.8 (±0.2)	0.21–1.39/**0.9 (±0.3)**	**0.8 (±0.1)**	**59.3 (±0.9)**	3.2 (±0.9)
8	0.73–1.46/0.9 (±0.3)	0.92–1.37/1.1 (±0.2)	1.0 (±0.1)	55.7 (±1.2)	2.6 (±1.0)

The bold numbers are significantly different from control values (last row of data) at *P* <0.05.

In the second experiment we have been tested if decreased CKX activity in seedling roots of T_1_ plants of STH7308 line silent for *HvCKX1* leads to increased root mass.

**Figure 3:**
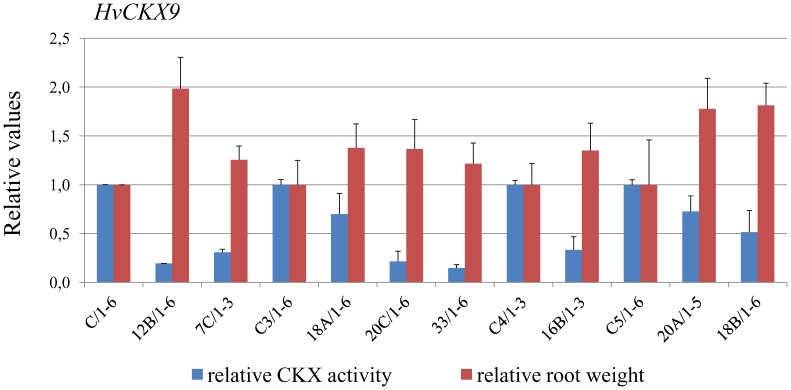
Relative activity of CKX enzyme and relative root weight in STH7308 lines silent for *HvCKX1* T_1_. C, C3, C4, C5 are internal control lines. * - significant differences at *P*<0.05.

To further test the suggestion from previous results that lower CKX activity might lead to greater root weight [8] in another genotype, plants from breeding line STH7308 silent for *HvCKX1* were obtained and tested. Relative activity of CKX enzyme and relative root weight were measured in 10 PCR positive T_1_ lines. The relative enzyme activity was reduced in 8 tested modified lines and ranged from 0.15 to 0.73 (Fig. 3). In contrast, the relative root weight was greater in silenced lines than in the control and ranged from 1.22 to 1.99 however the differences were significant only for two lines. These results indicate that decreased CKX activity in seedling roots of T_1_ plants would lead to increased root mass.

In the third experiment we have been tested if increased productivity and other phenotypic traits of lines silent for *HvCKX1* and *HvCKX9* are inherited through generations.

**Figure 4:**
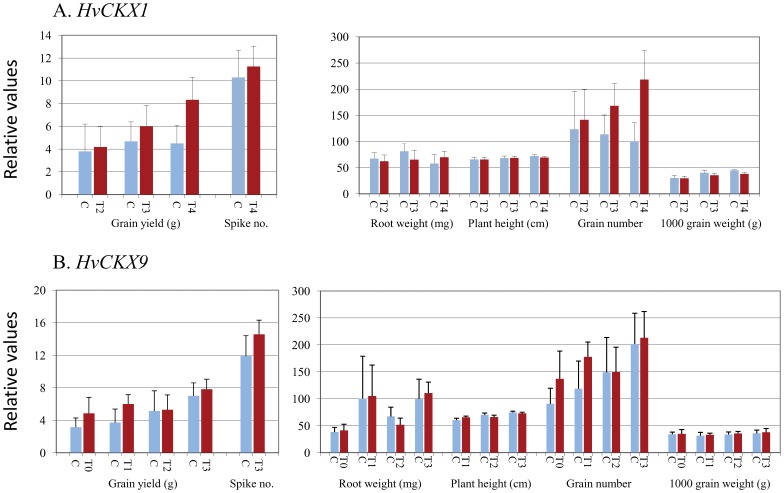
Analysis of inheritance of productivity and other phenotypic traits in subsequent generations. (A) T_2_, T_3_ and T_4_ lines silent for *HvCKX1* and (B) T_0_, T_1_ T_2_ and T_3_ lines silent for *HvCKX9*. * - significant differences at *P*<0.05.

The phenotypic characteristics assessed in mature plants from modified lines of subsequent generations included grain yield, root weight, plant height, grain number and 1000 grain weight (Fig. 4 a,b).

#### *HvCKX1* silenced lines

The first report on the *HvCKX1* silenced lines and analysis of their T_0_ and T_1_ generation was published previously [8]. Here we continue analysis of inheritance of productivity and other phenotypic traits in T_2_, T_3_ and T_4_ generations (Fig. 4 a). Grain yield was increasing in T_2_ and T_3_ generations of silenced lines and was significantly greater in T_4_ than in the control (transformed with the same vector without the silencing cassette), respectively 4.18 g (±1.8), 6.0 g (±1.8) and 8.33 g (±1.96) compared to 3.8 g (±2.4), 4.68 g (±1.7) and 4.5 g (±1.6). This trait was positively correlated with higher grain number in the T_2_ to T_4_ generations, which were 141.6 (±58) in T_2_ and 123.4 (±73) in the control, 168.3 (±43) in T_3_ and 113.7 (±37) in the control and 218.3 (±56) in T_4_ and 100.4 (±35) in the control lines. The grain number in T_4_ was significantly higher compared to the control. Root weight in silenced T_2_ and T_3_ lines was lower than in the corresponding control lines but greater in T_4_ lines, although the differences were not significant. Plant height was almost the same for silent and control lines in these three generations. 1000 grain weight was at a similar level in T_2_ and lower in T_3_ and T_4_. The mean values were 29.6, 35.6 and 38.2 in T_2_ to T_4_ and 30.2, 40.5, 45.1 in the corresponding control lines. 1000 grain weight in T_3_ and T_4_ was 12% to 15% lower than in the control. The greater grain number in T_4_ lines was correlated with higher spike number and lower 1000 grain weight, which finally resulted in significantly higher grain yield.

#### *HvCKX9* silenced lines

Although there were no significant effects, there were similar trends to *HvCKX1*. Grain yield was greater in T_0_ and T_1_ lines and only slightly greater in T_2_ and T_3_ compared to the control (Fig. 4 b). The data were as follows: 4.86 g (±1.9) in T_0_ and 3.15 g (±1.1) in the respective control; 6.0 g (±1.1) in T_1_ and 3.72 g (±1.6) in the control; 5.3 g (±1.8) in T_2_ and 5.15 g (±1.8) in the control; and 7.83 g (±1.2) in T_3_ compared to 7.0 g (±1.6) in the control. These data were correlated with greater (up to 50%) grain number than in the corresponding control in subsequent generations as well as slightly greater (2% to 5.9% above the control) 1000 grain weight. The data for root weight and plant height were variable in subsequent generations and did not show any tendency to increase or decrease.

Final grain yield in T_3_ was 11.9% higher than in the control and the components of these data were as follows: 5.7% greater grain number, 2.7% higher 1000 grain weight and 22.5% higher spike number (mean one more spike per silenced plant).

**Figure 5:**
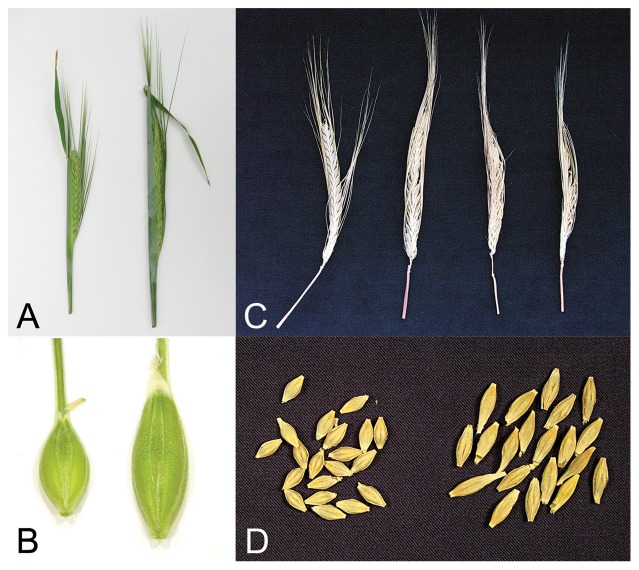
Phenotypes of typical and atypical *HvCKX9* silent lines. Typical silent (first left) and variant phenotype (right) of one selected atypical *HvCKX9* silent line showing long, immature kernels (A) and much bigger maturing grains (B), and not filled mature kernels (C) and only slightly bigger mature grains (D). Typical silent lines are phenotypically not distinguished from control lines.

Visually, phenotypes of *HvCKX1* and *HvCKX9* silent lines were indistinguishable from wild-type plants. However, in one transformation experiment with *HvCKX9* silencing, one line showed a variant phenotype (Fig. 5 a,b,c,d). Developing kernels were longer compared to the control and they formed much larger grains. The phenotype was inherited through the next two generations, but some of the kernels from T_1_ and T_2_ plants were empty or contained only a few seeds, so the total grain yield was lower than in the control. The activity of CKX enzyme in 7 DAP and 14 DAP kernels of T_1_ plants was less than in the control, but expression of *HvCKX9* in the same tissues was usually much greater than in the control (data not shown).

## Discussion

### Barley *HvCKX* gene family

According to the current NCBI database, 13 accession numbers of possible *HvCKX* genes were found and because of almost 100% homology between two of them, we can conclude that 11 members of the *HvCKX* family have already been identified. The first complete CDS of *HvCKX2* was downloaded in 2002 and its function was characterized based on heterologous expression in tobacco [16]. The second full-length cDNA, AK370106 [18], was found to be 99% homologous to JF495482 and named *HvCKX5* [17]. AK370106 was cloned from full-length cDNA libraries. JF495482 and ten other accession numbers were identified and named based on phylogenetic, molecular and comparative genomic analysis of the *CKX* gene family in the *Poaceae* [17]. Since rice homolog of barley AF490591 *CKX2* was not identified, primarily cloned *HvCKX2* was annotated by Mameaux et al. [17] as *HvCKX9*. According to this annotation the *HvCKX2* in our previous work [9], is renamed as *HvCKX9*. Respective, comparative analysis at the DNA, protein and genetic/physical map levels of *HvCKX2.1* and *HvCKX2.2*, suggested that the members are orthologous to wheat *TaCKX2*.3-5.

### Presumed function of selected *HvCKX* genes based on their expression profiles

The levels and the patterns of expression of five defined *HvCKX* genes in different tissues/organs of developing plants were studied to predict their potential biological functions and choose the most interesting one for further research. The expression patterns of the gene family members differ but were very similar for the same member in the two cultivars, which indicates their stable roles in barley. High levels of expression of *HvCKX1*, *HvCKX9*, *HvCKX4* and *HvCKX11* were observed in 14 DAP spikes compared to other tissues/organs, with expression of *HvCKX1* and *HvCKX4* reaching the highest levels. The level was 13–15 times higher than in seedling roots in the case of *HvCKX1* and around 30 times higher than in developed leaf tissue in the case of *HvCKX4*. Three gene family members of the five tested, *HvCKX9*, *HvCKX5*, and *HvCKX11,* showed high relative levels of expression in leaf tissues compared to others. Relative levels of expression exceeding 1.00 were also determined for *HvCKX4* in leaf tissues and seedling roots.

The same pattern of expression for *HvCKX1* was previously reported by Zalewski et al. [8] and is confirmed here. An expression profile of other selected *HvCKX* gene family members in different tissues/organs of developing barley plants by quantitative RT-PCR has not been done before. Previously, a semi-quantitative measure of expression of *HvCKX1* and *HvCKX9* (former *HvCKX2*) in grains, roots and leaves of barley showed the most intense signals for *HvCKX1* in grains and slightly less intense in 7-day roots and leaves [16]. This was not found in our analysis. In the case of *HvCKX9* the most intense signal of semiquantitative (not shown) and quantitative (this paper) expression was observed in leaves of seedlings, which was also not in agreement with our results obtained in two different cultivars. Some data about expression are available from EST databases (reviewed in Mameaux et al. [17]). For example, expression of *HvCKX1* was observed in callus, seedling leaf, and embryo sac 0–7 DAP. However, these data are difficult to compare, because of differences in thetissues tested and their developmental stages and most of all the unknown levels of expression. Additionally, there may be some differences between genotypes.

Some similarities in tissue and developmentally specific expression might be found between closely related *CKX* genes. According to phylogenetic and sequence analysis [17], [20], *HvCKX* are closely related to other members of the *Poaceae*. *HvCKX1* and *HvCKX2.1,2* are a grouping within clade Ia with *OsCKX1* and *OsCKX2* and are the closest homologues of *TaCKX1* and *TaCKX2.1-5* respectively [17]. Moreover, *HvCKX3* is the closest to *TaCKX3*, and *HvCKX4* to *TaCKX4*, but *HvCKX5* is the closest to *OsCKX5*. Some similarities in expression of these related genes were found. *TaCKX1* and *TaCKX2* were highly expressed in developing ovules/seeds with the peak at 2–4 DAP and the level of expression of the first was about 10 times higher than that of the second [20], as was observed for *HvCKX1* and *HvCKX9*. However, the relative expression of *TaCKX1* was also increasing during flag leaf development up to 28 DAP, which was not observed in barley. The expression of *TaCKX4* was very low in developing ovules/seeds, similar to the expression of *HvCKX4*. *OsCKX2* was predominantly expressed in the culms, inflorescence meristems and spikelets [7], which was similar, in the case of spikelets, to *HvCKX2* expression. These similarities in developmental and tissue-dependent expression of *CKX* genes between *H. vulgare*, *T. aestivum* and *O. sativa* supports the suggestion that phylogenetic analysis based on sequence similarity helps to predict common physiological functions.

### Patterns of expression of silenced *HvCKX* genes correlate with expected phenotypes

Our next step was to obtain lines silenced for selected *HvCKX* gene family members and assess correlations between patterns of expression in wild-type plants and the expected phenotype. Silenced lines, which are the equivalent of site-directed mutations, were obtained by a powerful laboratory tool, RNAi-based post-transcriptional gene silencing technology [29], [30]. The basic steps of the technology are construction of hpRNA type silencing cassettes [31] and their introduction to the genome of a plant by *Agrobacterium*-mediated transformation, which was established as the most suitable for silencing developmentally regulated genes [9]. Susceptibility of barley genotypes to this type of transformation is mainly restricted to the Golden Promise cultivar [32]–[34]. To compare results obtained for cv. Golden Promise with other genotypes, we used a breeding line, STH7308, had been shown to have a high rate of *Agrobacterium*-mediated transformation and was available for this type of research.

Since the family of *CKX* genes encodes tissue and developmentally specific CKX enzymes that irreversibly degrade cytokinins, reduces transcript level of the targeted *HvCKX* family member should lead to an increase in cytokinin in selected organs and developmental stages, as has already been documented in different species [7], [11], [35]. Consequently, changes at the molecular, physiological and phenotype levels should occur in the modified plants/lines. The most significant changes are expected in those tissues and organs which show a high transcript level and high enzyme activity. We have already documented that lower CKX activity in kernels and seedling roots of Golden Promise lines silenced for *HvCKX1* led to higher productivity in T_0_ and T_1_ plants and higher root weight in T_1_ seedlings [8]. In this research it was found that grain yield was higher in T_2_ and T_3_ generations of silenced lines and was significantly higher in the T_4_ generation, assuming that higher grain productivity was inherited. The main factor influencing grain yield was grain number and, less significantly, spike number. Similarly, reduced expression of *OsCKX2* in rice positively correlated with cytokinin accumulation in inflorescence meristems and increased number of reproductive organs resulting in enhanced grain yield [7].

A second characteristic of the silenced lines, greater root weight which was observed in T_1_ seedlings of cv. Golden Promise, was not inherited in subsequent generations. However, decreased CKX activity in seedling roots of T_1_ plants of the STH7308 line silenced for *HvCKX1* led again to increased root mass. This unstable effect of silencing might be dependent on relatively low expression of *HvCKX1* in seedling roots of wild plants, which is about 15 times lower than in 14 DAP kernels. Therefore the silencing might have less effect on this particular trait, especially in the next generations, where it might be compensated by coordinated regulation of other genes. Such regulation of multigene families *IPT*, *CKX*, *ZOG* and *GLU*, which regulate cytokinin synthesis and metabolism during flag leaf and reproductive development, was demonstrated in wheat [20]. Moreover, early development of seedlings are much more dependent on the features of the seeds, which might make this trait more unstable.

Earlier research with *A. thaliana* and tobacco transgenic lines overexpressing *AtCKX* genes, showed that, increased CKX activity led to reduced shoot growth and enhanced root growth. This was consistent with the hypothesis that cytokinins had opposite, regulatory functions in root and shoot meristems [3], [16] or they functioned as a negative regulator of root growth [36]. Our results were not consistent with the above conclusions. We think that the inconsistency is the result of differences in the applied approaches. The main difference is that the silencing was targeted to native *CKX* genes with spatial and temporal regulation and in consequence decreased enzyme level in the same pattern, in contrast to constitutive overexpression as applied in the *A. thaliana* and tobacco experiments [3], [16]. Moreover the inconsistency might be the effect of differences between the specificity of the species and their hormone trafficking, differences in temporal and spatial expression profiles of the tested *CKX* genes, compensation effect of other gene family members and differences between stage at which root development was assessed as a research material taken into research. Notably, the effect of greater mass of the seedling roots in barley which was coupled with decreased CKX activity was observed by us only in the early stages of root development and the differences between root systems of the two mature wild-type and silenced lines of two genotypes tested were not detectable (not showed).

Similar results of higher grain productivity in T_0_ and T_1_ after silencing of *HvCKX9* (former *HvCKX2*) were previously reported for several transgenic lines [9]. However, the main goal of that report was to show differences in silenced phenotypes obtained by two different methods of transformation. In this research we included all selected lines and documented that grain productivity was inherited through four generations, although the increase in grain yield in *HvCKX9* silenced lines was less than in *HvCKX1* silenced lines. Additionally, decreased CKX activity in leaves resulted in greater plant height in the T_1_ generation, but this silencing effect was not inherited through subsequent generations. This result was consistent with the result of mass of seedling roots in the case of *HvCKX1* silencing in cv. Golden Promise [8] and breeding line STH7308. In both cases the relative expression of wild-type *HvCKX1* in seedling roots and *HvCKX9* in leaves was rather low, which might influence the weakly inherited effect in T_1_ and no inheritance of the trait in subsequent generations. Slightly higher comparing with leaves wild-type *HvCKX9* expression in 14 DAP kernels resulted in a stable effect of silencing, showing trend of increased grain yield over generations. Substantially higher expression in developing kernels of wild-type *HvCKX1* resulted in significantly increased yield in T_4_ generation. Summing up these results, we can expect that silencing of *HvCKX4* might result in the highest rate of grain productivity and silencing of *HvCKX5* might cause greater plant height and that both traits would be inherited. This research is in progress.

The role of *CKX* in regulation of grain production was also reported for rice [7]. Reduced expression of *OsCKX2*, which is the closest homologue of *HvCKX1* and *HvCKX2.1,2*, caused cytokinin accumulation in inflorescence meristems and increased the number of reproductive organs, resulting in enhanced grain yield. The hexaploid wheat *TaCKX6*-D1 gene, which is the ortholog of rice *OsCKX2*, was documented as significantly associated with grain weight [10]. Higher grain weight was found in the haplotype with decreased expression of the gene in 8 DAP grains, which is similar to our results.

We have shown that the RNAi method is a useful tool to investigate gene function. Only one atypical phenotype among the several dozen obtained was found, showing much longer spikes with larger grains. This particular phenotype was inherited but the plants did not give higher yields under typical growing conditions, unlike other stable silenced lines showing a common phenotype.

## Conclusions

We have demonstrated that the levels and patterns of expression of *HvCKX* gene family members in various tissues/organs of developing barley plants were specific to the family member and were very similar between the two genotypes tested. Reduction of expression of the selected *HvCKX* gene family member by RNAi silencing reduced CKX enzyme activity, especially in those tissues/organs which showed the highest expression in wild-type plants. The documented effect of RNAi silencing of *HvCKX1* in developing spikes resulted in enhanced grain yield, and the trait was inherited through four generations. The *HvCKX9* silenced lines showed similar trend of increasing grain yield over the generation, however the differences were not significant. The increased yield was influenced by a greater number of seeds and bigger number of spikes. The increase in productivity was significantly greater in *HvCKX1* silenced plants and these showed a greater relative expression of *HvCKX1* in developing kernels of wild-type plants compared to the expression of *HvCKX9*. Silencing of a gene family member that showed a lower expression in a particular organ caused less intense phenotype changes in the T_1_ generation, and which were not inherited through subsequent generations. We showed that the patterns and levels of expression of *HvCKX* gene family members in developing barley plants are indicators of their role in growth and reproductive development.
